# Satisfying basic psychological needs through a recreational sports programme for people with intellectual disability: human growth and adapted sport in focus

**DOI:** 10.3389/fpsyg.2024.1470411

**Published:** 2024-12-05

**Authors:** Nerea Crespo-Eguílaz, Leyre Gambra, Apolinar Varela, Raúl Fraguela-Vale

**Affiliations:** ^1^Pediatric Neurology Unit, Clinic University of Navarra, Pamplona, Spain; ^2^Faculty of Education, Universidad Internacional de La Rioja, Logroño, Spain; ^3^Faculty of Education and Psychology, University of Navarra, Pamplona, Spain; ^4^Faculty of Education, University of A Coruña, A Coruña, Spain

**Keywords:** basic psychological needs, adapted sport, human growth, intellectual disability, self-determination theory

## Abstract

**Introduction:**

Self-determination theory (SDT) highlights the importance of satisfying people’s basic psychological needs (BPN) (autonomy, competence and relatedness) in order to ensure their personal growth and wellbeing. In this regard, sport and physical activity (PA) have been shown to offer significant health benefits, particularly in the case of people with intellectual disability (ID), among whom the benefits are even more noticeable owing to their low levels of PA, sedentary lifestyle, limited opportunities to exercise, and consequently lower quality of life. The aim of this study is to assess the impact of the Más Que Tenis (“More Than Just Tennis”) inclusive recreational sports programme on the satisfaction of BPN among athletes with ID, taking into account factors such as age, gender and type of activity.

**Methods:**

The sample for the study comprised 50 athletes (68% male) with ID (IQ: *X* = 54.33; sd = 13.43), aged 17–54 years. Data were collected using the Spanish version of the Basic Psychological Needs in Exercise Scale (BPNES), Kaufman Brief Intelligence Test (K-BIT), Satisfaction With Life Scale (SWLS), multidimensional AF5 self-concept scale, Vineland-3 Scale, and MABC-2 observation checklist.

**Results:**

High levels of satisfaction of all three BPN, with autonomy scoring lowest of the three. Gender was found to be a significant predictor, with men scoring higher in all BNP. Participants reported positive perceptions in relation to physical self-concept and satisfaction with life. In terms of adaptive behaviour, deficits were detected in relation to communication and daily living skills, but not in relation to interpersonal skills. With respect to motor behaviour, satisfaction of BPN was found to correlate more with dynamic environments than with static ones. The findings indicate the effectiveness of the Más Que Tenis programme in satisfying the BPN of people with ID. Participants reported improved physical skills, competence, social integration and interpersonal relations, though also insufficient autonomy in relation to choosing what exercises to do.

**Conclusion:**

Satisfaction of BPN through the programme was found to correlate positively with athletes’ satisfaction with life and adaptive development, leading to improved personal and neuropsychological wellbeing.

## 1 Introduction

Much research on physical activity (PA) draws on self-determination theory (SDT) to examine factors such as motivation and the psychological processes associated with wellbeing (Deci and Ryan, 1985, 2002; Ryan and Deci, 2000; Franco et al., 2017; Moreno et al., 2008; Ntoumanis and Standage, 2009). SDT is a macro-theory of motivation and personality that comprises five sub-theories, including that of basic psychological needs (BPN) (Stover et al., 2017). According to BPN theory, there are three human needs—autonomy, competence and relatedness—that are innate, universal and essential, and therefore must be met in order to ensure optimal health and functioning. The need for autonomy refers to a person’s self-perception as the causal locus of their actions, and that those actions are not determined by external factors (Ryan and Deci, 2007). Perception of autonomy is a positive predictor of satisfaction of the other two BNP, which in turn predicts greater self-determined motivation (Standage et al., 2006). The need for competence refers to the ability to perform tasks of varying complexity. It is positively associated with good stress regulation, self-esteem and wellbeing, and negatively associated with depression, anxiety and low self-esteem (Parfitt et al., 2009). Finally, relatedness is the extent to which a person feels securely part of a group or connected with others within his or her social context. BPN may thus be seen as psychological mediators that influence intrinsic and extrinsic motivation and demotivation, and satisfaction of those needs may be seen as an essential component of human wellbeing and development (Ryan and Deci, 2017; Vansteenkiste et al., 2020).

Satisfaction of BPN among people with Intellectual Disability (ID) has been shown to be affected by their living, working and educational environments (Akkerman et al., 2018; Wehmeyer and Bolding, 1999), where self-determination, autonomy and satisfaction predict improved wellbeing and educational outcomes. SDT is particularly relevant to studies with populations with ID because it focuses on satisfying BPN fundamental to wellbeing and intrinsic motivation, independent of cognitive abilities. This theory provides a framework for understanding how people with ID can develop a sense of control and self-direction, which is crucial for their social inclusion and personal development. However, it assumes the limitation that the application of SDT may require specific adaptations to consider the cognitive and communication barriers present in this population. Among other adaptations, it is necessary to develop assessment instruments that are explicitly adapted to them (Katz and Cohen, 2014). Despite these limitations, studies have found SDT valid and applicable to people with intellectual disabilities (Behzadnia et al., 2022; Emond-Pelletier and Joussemet, 2017; Frielink et al., 2018). American Association on Intellectual and Developmental Disabilities (2010) views self-determination as a key goal for individuals with ID.

Compared to the general population without disabilities, individuals with ID encounter more discrimination and social marginalization related to gender and social class, fewer employment prospects, and limited access to education, sports, and leisure activities (Stevenson, 2009; Van Lindert et al., 2023). Despite the proven advantages of adapted physical activity (APA) and sports for individuals with ID, there are limited tailored sports opportunities for people with these needs. People with ID often experience low physical activity, poor diet, limited participation in sports, and high levels of sedentary behaviour, leading to poorer physical, mental, and social health, as well as reduced life expectancy (Borland et al., 2020; Hansen et al., 2023; Robertson et al., 2000; Temple et al., 2006; Wouters et al., 2019). In recent decades, sports for individuals with ID have grown significantly, driven by greater visibility, social recognition, and funding for Paralympic sports (Leardy and Sanz, 2018). However, there is a distinction between Paralympic sports and non-competitive activities focused on recreation rather than performance (Pérez-Tejero, 2024; Solves, 2018). Instead, the focus is on enabling people with ID to participate in sports activities that enhance their wellbeing and quality of life through APA or sports. In these instances, sports and recreational programmes designed to promote the inclusion and integration of individuals with ID do not enjoy the same level of visibility as Paralympic or competitive sports. Consequently, more research is needed on factors affecting quality of life for people with ID (Xiao et al., 2024) and on sports and leisure programmes for this population (Díaz et al., 2019; Evans et al., 2018). Most studies on sport participation in people with ID have focused on competition, performance, barriers, facilitators, and coaches (Mendoza and Gamonales, 2024). However, research on the impact of recreational sports programmes for people with ID remains limited (Arbour-Nicitopoulos et al., 2022; Díaz et al., 2019; Maenhout and Melville, 2024; Mendoza and Gamonales, 2024; Pinilla and Pérez-Tejero, 2017; Pérez-Tejero, 2024).

Researchers agree on the physical, intellectual and psychosocial benefits of adapted physical activity (APA) and sport for people with ID (Bondar et al., 2020; Cabeza-Ruiz et al., 2021; Chinga and Martínez, 2020; Collins and Staples, 2017; Gámez-Calvo et al., 2022; Gamonales et al., 2021; Jacob et al., 2023; Pérez-Tejero and Ocete, 2016; Van Lindert et al., 2023). The relationship between neuropsychology, wellbeing, sports, and intellectual disability is documented in the literature. For example, Eime et al. (2013) highlight the psychological and social benefits of sports participation for mental health and wellbeing, including improved self-esteem and socialisation, crucial for people with ID; Arellano et al. (2024) and Varela et al. (2025). emphasise benefits for athletes with ID, such as psychological wellbeing, a sense of community, social inclusion, autonomy, motivation, and physical gains from recreational sports; White et al. (2017) found that a soccer programme for people with ID enhanced their social, emotional, and personal development, along with enjoyment and team belonging; Mckinnon et al. (2022) demonstrated that sports foster physical improvements, body image, social belonging, leadership, friendships, autonomy, and life skills transferable to daily life. Maenhout and Melville (2024) highlight how physical activity promotes social connectedness, personal growth, and wellbeing for people with ID; Shapiro and Martin (2014) noted that motor competence in sport improves self-esteem, friendships, and reduces loneliness; Tomé et al. (2024) found that physical activity enhances autonomy and daily living skills in individuals with ID. Additionally, research shows that sports enhance executive functions, cognitive flexibility, memory, brain activation, and inhibitory control, stress and anxiety, boost emotional wellbeing and mental health (Komenda et al., 2023; Martín-Rodríguez et al., 2024; Liang et al., 2022; Liu et al., 2024; Xiao et al., 2024). Despite the universal nature of BPN, little research has focused on BPN among athletes with ID. Most studies have centred on neurotypical subjects, with few quantitative studies exploring BPN models or satisfaction through sports and physical activity in people with ID. It is crucial to examine how this theory applies specifically to populations like individuals with ID in Spain.

Research findings indicate that coaching behaviour can be a predictor of satisfaction of BPN among athletes with ID (Komenda et al., 2022), and that more controlling behaviours have a negative impact in this regard (Hu et al., 2023). In support of this, Farrell et al. (2004) identify a relationship between self-determination and intrinsic motivation among athletes with ID, based on a qualitative study involving 38 interviews. Shangraw (2017) explores how coaches can support their athletes’ feelings of autonomy, competence and relatedness, while Pires et al. (2021) descriptive analysis examines the correlation between improvement-and reinforcement-based coaching behaviours and high levels of autonomous motivation, satisfaction with life and positive affect. In relation to support models—including coaching—and the applicability of SDT in this regard, Frielink et al. (2018, p. 45) conclude that “SDT shows potential as a guide towards enhancing subjective wellbeing and thus quality of life of people with ID with a mild to borderline level of functioning through support focused on autonomy.”

Studies show that autonomy, competence and relatedness influence persistence in PA among adolescents without disability. According to Babic et al. (2014), higher perceived competence, fitness, appearance and physical self-concept is associated with higher levels of PA, though it is unclear whether PA improves physical self-concept or vice versa (De la Torre Cruz et al., 2018). Fernández-Bustos et al. (2019) study on the relationship between physical self-concept and PA among adolescents using the physical self-concept subscale of the AF5 self-concept questionnaire (García and Musitu, 2014) shows a significant association between physical self-concept and PA, with sex and age identified as key mediating factors; according to Reigal et al. (2014), this association is higher among athletes who participate in sport or other PA on a regular basis. Relatedness and socialisation (connection and social acceptance) are, likewise, positive predictors of participation in PA (Sánchez and Núñez, 2007; Peres et al., 2012), as are social support networks and emotional ties to family and friends (Balaguer et al., 2008; Cheng et al., 2014). In relation to people with ID, Pan and Davis (2019) report positive impacts on physical self-concept from participating in sports activities; here physical self-concept refers not just to physical appearance but also fitness, strength and sports competence (Goñi et al., 2004).

The rationale for the present study is twofold. Firstly and more generally, the research attempts to fill the gap in the literature regarding the satisfaction of BPN through sport in relation to people with ID. Secondly and more specifically, this study diverges from the more usual focus of research in this area on athletes with ID involved in competitive sporting programmes such as the Special Olympics, in order to examine questions of human growth and adapted sport in relation to a non-competitive recreational sports programme for people with ID with diverse levels of sports skills. In doing so, the study expands and diversifies the existing knowledge about satisfaction of BPN through sport for people with ID.

The general aim of the study is to assess the contribution of the *Más Que Tenis* (Más Que Tenis—“More Than Just Tennis”) recreational sports programme to the satisfaction of BPN among athletes with ID, and to analyse the relationship between satisfaction of BPN and physical self-concept, motor behaviour, satisfaction with life, adaptive behaviour and gender.

Based on this general aim, the specific aims of the study are:

1. To assess satisfaction of BPN (autonomy, competence, relatedness) among participants in the Más Que Tenis programme.
2. To analyse the relationship between satisfaction of BPN, physical self-concept and satisfaction with life among athletes with ID.
3. To ascertain the relationship between satisfaction of BPN, motor behaviour and adaptive behaviour among athletes with ID.
4. To examine the role of gender as a predictor of satisfaction of BPN through the programme.

Based on the specific objectives above, four working hypotheses are proposed.

1. *Hypothesis 1 (H1):* The satisfaction of the needs of the BPN is expected to be high, and the need for relatedness will be of particular importance. Since this is a voluntary participation leisure programme, it is expected that the satisfaction of the BPN will be high and that the relationship with peers (relatedness) will be a strong point, to the detriment of the satisfaction of the need for competence that is more relevant in competitive and high-performance sports contexts.
2. *Hypothesis 2 (H2):* A positive and significant relationship is predicted between BPN and physical self-concept, as well as between BPN and life satisfaction.
3. *Hypothesis 3 (H3):* A positive relationship between BPN and motor behaviour is expected.
4. *Hypothesis 4 (H4):* Significant differences in favour of boys in the satisfaction of all BPNs within the programme are expected.

## 2 Materials and methods

The study uses a quantitative cross-sectional methodology, based on the following elements.

### 2.1 Más Que Tenis recreational sports programme for athletes with ID

Más Que Tenis is a recreational sports programme run by the Rafa Nadal Foundation in collaboration with Special Olympics Spain. The main aim of the programme is to promote tennis among people with ID as a way of encouraging healthy habits and active leisure, improving motor skills, socialisation and wellbeing, promoting values associated with sport, and increasing the inclusion and visibility of people with ID. Training sessions are held in the mornings (except in Madrid and Aragon) 1–2 times a week, and last 60–90 min each. The exercises proposed in the programme are divided into: introductory tennis content focused on ball + racket: basic grip, types of strokes; ball + racket + net: parabolic trajectory of the ball; ball + racket + net + playing area: force control; Technical and tactical exercises like forehand, backhand, volley, underhand serve, overhand serve, global game practise (collaboration 1 + 1, opposition 1 × 1…) or real game situations (actual tennis competitions 1 × 1 or 2 × 2); Physical contents like General Dynamic Coordination (Skills): movements, jumps, turns, balances or Specific Dynamic Coordination (Dexterities): throws, catches, strokes or bounces. These exercises vary according to the level of the athletes, their motor skills and cognitive demands. The sports facilities where the sessions take place are all separate from the occupational centres, associations, clubs, etc. where the athletes usually spend their days. The programme’s coaching and coordinating staff comprises a diverse mix of qualified intellectual disability professionals, including pedagogues, physical education specialists, and graduates in primary school teaching and physical activity and sports science, with experience in day and occupational centres, associations and clubs. Staff members also include qualified tennis instructors, whose role is limited to teaching the athletes how to play tennis, and volunteers. All of the athletes in the study have been involved in the programme for 2–10 years.

### 2.2 Participants

At the time of the study, there were 22 Más Que Tenis schools in operation across different regions of Spain, with a total of 250 participating athletes. The sample for the study comprised 50 athletes (34 male, 16 female) from 6 of those schools (27.27%) in 6 different regions (Aragon, Andalusia, Balearic Islands, Galicia, Madrid, Valencia), selected using a non-probability sampling method based on ease of access to the schools. The age range of participants was 17–54 years (*X* = 33.68, standard deviation-sd = 10.14), with 78% aged over 25.94% live with family members, 2% in residential settings, and 4% independently. The standard formula for sample size calculation has been used, considering a 95% confidence level and a 5% margin of error. The minimum sample size necessary to adequately represent the population of people with intellectual disabilities who practise sports (approximately 1.4% of the total population) is 21 athletes to represent this population within the mentioned statistical margins.

All participants have an Intelligence Quotient (IQ) significantly below the neurotypical average, as assessed by the K-BIT Scale (Kaufman and Kaufman, 2000), ranging from borderline to moderate (see Table 1), with an average score of 54.33 (sd = 13.43). All participants also have adaptive behaviour deficits, as assessed using the Vineland-3 Adaptive Behaviour Scale (Sparrow et al., 1984), as detailed in the Results section.

**Table 1:** Number of participants and percentage of participants by category based on IQ.

Participants *n* = 50	*n*	%
Total IQ ranges		
Borderline: 84–70	7	14%
Mild intellectual disability: 69–50	25	50%
Moderate intellectual disability: 49–35	18	36%

The diagnostic criteria of the *Diagnostic and Statistical Manual of Mental Disorders* (DSM-5) include impairment of general mental abilities (IQ < 70) and of adaptive functioning in conceptual, social and practical domains (American Psychiatry Association, 2014). Participants with borderline IQ scores (70–84) have been included in the sample (*n* = 7, 14%) on the basis that, while their mental ability exceeds the criterion for intellectual disability, their deficits in relation to adaptive functioning mean they still require significant supports to cope with different aspects of everyday life. For the purposes of this study, therefore, they have been classed as athletes with ID (Frielink et al., 2018).

### 2.3 Tools and procedures

The data for the study were collected using the following research instruments.

#### 2.3.1 Kaufman brief intelligence test

The Kaufman Brief Intelligence Test (K-BIT) (Kaufman and Kaufman, 2000) is used in clinical, educational and research settings as a quick, reliable measure of general intelligence. K-BIT consists of two sub-tests: verbal knowledge, which assesses word knowledge through the identification of pictures and verbal analogies; and matrices, which measures non-verbal intelligence and perceptual and abstract reasoning based on the subject’s ability to complete visual analogies and understand relationships. The test provides scores for total, verbal and non-verbal IQ.

#### 2.3.2 Basic psychological needs in exercise scale

The study uses the Spanish version (Moreno et al., 2008) of the Basic Psychological Needs in Exercise Scale (BPNES) (Vlachopoulos and Michailidou, 2006). The scale consists of 12 items divided across three domains: autonomy, competence, and relatedness. The language of the scale was adapted to make it clear to participants that the items referred to activities related to the Más Que Tenis programme, as reflected in Table 2 below. As an additional adaptation, face pictograms were added to items to represent the agreement continuum visually as well as verbally to make it easier for participants to answer. Answers were scored according to a Likert scale ranging from 1 (totally disagree) to 5 (totally agree). Cronbach’s alpha scores for the three domains in relation to participation in PA outside of the programme were: autonomy: 0.69; competence: 0.81; and relatedness: 0.78.

**Table 2:** Results obtained from BPNES.

Domains and items		Disagree	Neither agree nor disagree	Agree
		*n* (%)	*n* (%)	*n* (%)
Autonomy				
	Regarding the exercises in my tennis class:			
1	The exercises match my interests	4 (8%)	4 (8%)	42 (84%)
4	The exercises match what I want to work on	2 (4%)	3 (6%)	45 (90%)
7	The way I exercise matches my desires	0	9 (18%)	41 (82%)
10	I have the opportunity to make choices about the way I exercise*	17 (34%)	9 (18%)	24 (48%)
Competence				
	In my tennis classes:			
2	I have improved	3 (6%)	4 (8%)	43 (86%)
5	I can do the exercises correctly	3 (6%)	2 (4%)	45 (90%)
8	I do the activities very well	3 (6%)	4 (8%)	43 (86%)
11	I can meet the demands of the physical activities	8 (16%)	6 (12%)	36 (72%)
Relatedness				
	Regarding my tennis classmates:			
3	I feel comfortable doing exercise with my classmates	2 (4%)	3 (6%)	45 (90%)
6	My relationships with my classmates are friendly	1 (2%)	4 (8%)	45 (90%)
9	I can communicate openly with my classmates	3 (6%)	2 (4%)	45 (90%)
12	I feel very comfortable with my classmates	4 (8%)	1 (2%)	45 (90%)

Number of participants and frequency of responses.

^*^ Friedman test: results for item 10 differ significantly from the rest of the items in the category (*χ*^2^ = 45.92; *p* < 0.001). No significant differences detected in relation to Competence (*χ*^2^ = 4.94; *p* > 0.05) or Relatedness (*χ*^2^ = 0.65; *p* > 0.05).

BPNES was chosen over Frielink et al. (2018) adapted instrument for measuring satisfaction and frustration of BPN among people with ID owing to the length of the latter (24 items) and the fact that it does not focus specifically on sports activities. In addition, BPNES has already been successfully used in other studies involving subjects with a similar IQ (equivalent to a mental age of 8–9 years minimum) (Prado-Botana et al., 2023; Navarro-Patón et al., 2018), demonstrating its validity among study populations with these characteristics. Finally, the positive reliability scores highlighted in the previous paragraph indicate a good understanding of the scale among those tested.

BPNES scores depend on a wide range of factors, including gender, age, type of PA and the way PA is organised (Lamoneda and Huertas-Delgado, 2019). With respect to gender, studies show that male athletes tend to score higher than females, in some instances across all three domains (Brunet and Sabiston, 2009), and in others, in relation to autonomy and competence (Gómez-Rijo et al., 2014).

BNPES scores have been shown to be a significant predictor of PA. In studies involving adolescents, participants with positive scores in all three dimensions (i.e., who perceive themselves as autonomous, competent, and having a good relationship with their peers) showed greater intrinsic motivation and self-determination, which in turn led to greater persistence in PA and improved health outcomes (Almagro et al., 2011; Sánchez-Oliva et al., 2013; Sicilia-Camacho et al., 2014).

#### 2.3.3 Satisfaction with life scale

The Satisfaction with Life Scale (SWLS) (Pons et al., 2002) measures overall individual satisfaction with life based on five items related to personal goals and perceived wellbeing. It is a valuable tool for clinical assessment of subjective wellbeing. Cronbach’s alpha for the scale was calculated to be 0.79.

#### 2.3.4 Physical self-concept according to the multidimensional AF5 self-concept scale

The multidimensional AF5 self-concept scale by García and Musitu (2014) measures academic, social, emotional, family and physical self-concept. The full scale comprises 30 items, six per domain, with a response range of 1–99. AF5 is one of the most commonly used instruments among Spanish-speaking samples (Lila et al., 2000; Pellerano et al., 2006). Its domain structure has been assessed using both exploratory factor analysis (García and Musitu, 2014) and confirmatory factor analysis (García et al., 2011; García et al., 2006).

Taking as a reference what is indicated by the authors themselves, this study focuses on the analysis of self-concept, more specifically on physical self-concept. For this reason, in this study, only the items related to physical concept were used: “I’m good at sport”; “I like myself physically”; “I’m attractive”; “People ask me to play with them”; “I look after myself physically”; and “I think I’m elegant.” As before (BPNES escale), face pictograms were added to items to represent the agreement continuum visually as well as verbally. Cronbach’s alpha for the physical self-concept section of the scale was calculated to be 0.96.

#### 2.3.5 Vineland-3 adaptive behaviour scale

The Vineland-3 Adaptive Behaviour Scale (Sparrow et al., 1984) was completed by participants’ parents and primary carers, as those best informed to do so, based on adaptive behaviour in four domains: communication (receptive language, expressive language, and writing); daily living skills (personal, domestic, and community); socialisation (interpersonal relationships, play and leisure, and coping skills); and motor skills. The final domain was excluded from this research as the normative range is restricted to 0–9 years. Items for the other domains include: “Looks after him/herself”; “Goes to familiar places on his/her own”; and “Can do skilled jobs.” Cronbach’s alpha values for the study were calculated as: communication: 0.78; daily living skills: 0.82; and socialisation: 0.89.

#### 2.3.6 Observation checklist from the MABC-2 movement assessment battery

The behavioural observation checklist (BOC) from the MABC-2 Movement Assessment Battery (Henderson et al., 2007) provided qualitative data from family members and coaches regarding participants’ motor skills and behaviour in everyday contexts, and the impact of any motor problems on their everyday activities, academic learning, recreational activities and social interactions. The checklist is divided into two sections: stable-predictable, focusing on participants’ behaviour and performance in a controlled environment (BOC-a); and dynamic-unpredictable, examining participants’ performance of physical activities and games involving fast movement and changes (BOC-b). Cronbach’s alpha values for the study were calculated as: BOC-a: 0.81; and BOC-b: 0.83.

The first step in the research process was to contact the people in charge of the Más Que Tenis programme and inform them of the aims and procedure of the research. Family members were informed about the research and its implications through a series of talks, and by letter in the case of those unable to attend. An information pack and informed consent forms were sent by email to schools for distribution among parents and legal guardians, and signed forms returned to coaches and forwarded to the research team. Following confirmation of consent, the schools were sent all of the data instruments and instructions on how to use them.

Once the sample for each school was established, the research instruments were applied according to a mixed postal and in-person methodology, according to limitations of space, time, finance and human resources. In the case of the MABC observation checklist (with separate versions for staff and family members) and the Vineland-3 Adaptive Behaviour Scale, family members and project staff were sent the instruments and instructions on how to use them on an individual basis. Older family members were assisted where necessary by school staff, who were already familiar with the athletes from their years of working with them in occupational and day. To respond to the two scales and given their breadth of items, the time to complete the two instruments was about 60 min.

The same system of individual letter post was used in the case of BPNES, SWLS and AF5, which were completed by the athletes with ID. However, in this instance, respondents were allowed to receive support to understand the items and examples featured in the instruments and also to fill them in by writing on the paper. Members of the research team were also available to clarify any doubts or questions, and, in one instance, to administer the instrument with the help of day centre staff. Due to the special characteristics of the athletes and the need for support in understanding and responding to some of the items, the average time to respond to the three instruments as a whole was about 30 min.

The in-person methodology used in relation to K-BIT involved data collection visits by 1–3 members of the research team depending on the participants’ availability. Tests were administered based on the instructions included in the test manual, and lasted 30–50 min, depending on each participant. Responses were provided on an individual basis with no assistance or support.

#### 2.3.7 Statistical analysis

The data collected were subjected to descriptive and inferential analysis using tools from the IBM SPSS Statistics 28.0 package (2017, IBM Corp., Armonk, NY, USA). Descriptive analysis used measurements of frequency, dispersion and central tendency, while inferential analysis used non-parametric statistics because the conditions of normal distribution and equal variance were not met. Magnitude and significance of differences between variables were calculated using the Friedman test, and pairwise comparisons were made using the Wilcoxon rank test. Correlations between variables were assessed using Spearman’s rho, gendered comparisons were carried out using the Mann–Whitney U test, and comparisons by age and IQ were measured using the Kruskal–Wallis test. A 0.05 level of significance was used.

### 2.4 Ethical considerations

Ethical approval for the study, consent form and questionnaires was obtained in advance from the University of Navarra Ethics Committee (reference number: project 2021.200; date of approval: 09/12/2021). Prior to data collection, participants were informed of the voluntary, anonymous nature of their involvement in the study, and provided with an easy-to-read informed consent form in Spanish to sign. The participants’ legal guardians received a letter containing the research plan and aims, together with information about their right to revoke consent; this provision was approved by the University of Navarra Ethics Committee as a legal requirement under Spanish law.

Research transparency is one of the core principles of this study. Consequently, all activities related to the design, planning and implementation of the study have been conducted in accordance with the Spanish Protection of Personal Data and Digital Rights Act (LOPD) 3/2018. The content management tool provided by the University of Navarra has been used to ensure the ethical collection and storage of all research data, in accordance with the European Data Protection Regulation (EU) 2016/679. Finally, participants were informed by letter regarding the research plan and provided with an informed consent form, information meetings were held with staff and family members to explain their role in the study, and all personal data were treated anonymously and confidentially, in accordance with the globally recognised code of ethics of American Educational Research Association (2011).

## 3 Results

The results below are presented in four blocks, corresponding to the four specific aims of the research.

### 3.1 Satisfaction of basic psychological needs in the Más Que Tenis recreational sports programme

The results of the data collected using BPNES show a high level of satisfaction of all three BPN among participants.

In relation to autonomy, most participants reported that the exercises match their interests (84%) and what they want to work on (90%). In contrast, the level of satisfaction of the need for autonomy reported in relation to making choices about how to do the exercises (item 10) was significantly lower, with only 48% in agreement with the statement and 34% in disagreement. This indicates that choosing how to do the exercises is an area in which participants perceive less independence.

Regarding competence, the majority of participants reported improved skills (86%), the ability to do the exercises correctly (90%), and success in meeting the demands of the physical activities (72%).

In terms of relatedness, participants reported that they feel comfortable doing exercise with their classmates, have a friendly relationship with classmates, can communicate openly, and feel very comfortable in class (90% agreement in all cases).

As the results in Table 3 illustrate, the scores for all three BPN may be classed as high, with values in excess of 4 on a 5-point scale. To examine the differences between the variables, pairwise comparisons were made using the Wilcoxon rank test, the results of which showed no differences (*p* > 0.05) between autonomy and competence, but significantly lower scores for both than for relatedness (*p* < 0.05). This indicates that, while participation in the Más Que Tenis programme satisfies all BPN, it does so most significantly in relation to relatedness.

**Table 3:** Variables for BPNES, AF5 and SWLS (*n* = 50).

Test	Variables	*M*	SD	Age	IQ
				*χ^2^ (p)*	*χ^2^ (p)*
BPNES	Autonomy	4.08	0.76	21.15 (0.572)	24.98 (0.520)
	Competence	4.23	0.74	27.31 (0.243)	18.82 (0.844)
	Relatedness	4.43	0.64	29.18 (0.174)	20.42 (0.771)
AF5	Physical self-concept	59.04	36.98	14.19 (0.912)	33.09 (0.159)
SWLS	Life satisfaction	4.14	0.84	20.79 (0.594)	19.33 (0.822)

Comparison of scores for each variable by age and IQ.

BPNES, Basic Psychological Needs in Exercise Scale; AF5, AF5 self-concept scale; SWLS, Satisfaction With Life Scale; IQ, Intelligence Quotient; *M*, mean; SD, standard deviation; *χ*^2^, Chi-Square; *p*, bilateral signification. Comparison between groups by age and IQ (Kruskal Wallis).

The lack of any significant differences in relation to age or IQ (Table 3) indicates that perception of satisfaction of BPN across the sample is not affected by either variable.

### 3.2 Satisfaction of basic psychological needs and personal wellbeing

The results for the AF5 self-concept scale show a generally positive physical self-concept among participants, but also considerable variability between answers (Table 3). For example, the statement in item n°1 “I’m good at sports” was scored higher than 50 by 80% of participants, and 68% indicated “I like the way I am physically” (item 2). However, the results of the comparative Wilcoxon test analysis show that the latter perception differs significantly from the perceptions reported in relation to the statements “I am an attractive person” (item 3: *Z* = −2.4; *p* < 0.05), “I take care of myself physically” (item 5: *Z* = −3.31; *p* < 0.01) and “I consider myself elegant” (item 6: *Z* = −2.21; *p* < 0.05). These differences suggest that, while many of the participants have positive feelings regarding their sporting ability and physical appearance, their self-perception in terms of attractiveness and physical self-care is significantly less favourable.

In relation to SWLS, most participants reported a favourable perception of their personal circumstances and adequate satisfaction with their lives. Most also consider that they have achieved important things in their lives, and would not be inclined to change much if they could live their lives over. These results indicate a generally high satisfaction among participants with different aspects of their lives, including their current circumstances, achievement of important goals, and acceptance of their life path. No significant differences were observed between the different items (Friedman test: *χ*^2^ = 8.49; *p* > 0.05), indicating consistency across the sample in relation to participants’ general perception of satisfaction with life.

Table 4 shows the correlations between the different variables. As in other studies, highly significant correlations between BPN were found in all instances. BPN were also associated with life satisfaction, while their correlation with physical self-concept was more moderate, with significant values in relation to autonomy and competence, but not relatedness. These results highlight the connection between satisfaction of BPN and participants’ personal wellbeing.

**Table 4:** Correlations of basic psychological needs, physical self-concept and life satisfaction (*n* = 50).

	Autonomy	Competence	Relatedness	Physical self-concept	Life satisfaction
A		0.743***	0.734***	0.328*	0.599***
C			0.778***	0.364*	0.706***
R				0.248	0.666***
PSC					0.296*
LS					

Spearman’s Rho. *p < 0.05, **p < 0.01, ***p < 0.001. A = BPN Autonomy; C = BPN Competence; R = BPN Relatedness; PSC = Physical Self-Concept; LS = Life Satisfaction.

### 3.3 Satisfaction of basic psychological needs and adaptive behaviour

The results of the descriptive statistics analysis of the Vineland-3 Adaptive Behaviour Scale show that participants’ performance is significantly below the neurotypical average, indicating marked deficits in key areas of adaptive functioning (see Table 5).

**Table 5:** Descriptive statistics of domains (communication, daily living skills, and socialisation) according to the Vineland-3 scale.

	Descriptive statistics *x (ds)*	Comparison between sub-tests *Z (p)*		Comparison between domains *Z (p)*	
				DLS	S
**Communication-C**	21.78 (6.33)	CEx	CW	−5.84***	−5.58***
Receptive (CR)	1.14 (0.41)	−5.44***	−6.16***		
Expressive (CEx)	4.02 (2.33)		−2.71**		
Written (CW)	3.01 (2.71)				
**Daily living skills-DLS**	45.36 (13.79)	DLSd	DLSc		−0.175
Personal (DLSp)	2.28 (1.05)	−5.98***	−5.73***		
Domestic (DLSd)	7.46 (3.65)		−0.96		
Community (DLSc)	7.04 (4.06)				
**Socialisation-S**	45.07 (18.28)	SPL	SC		
Interpersonal (SI)	10.45 (3.65)	−6.05***	−5.67***		
Play and leisure (SPL)	3.12 (1.99)		−5.86***		
Coping (SC)	6.82 (3.23)				

Comparison of performance by sub-test and domain.

Vineland-3 scale domains by IQ score (mean 100; standard deviation 15) and domain sub-tests in scaled scores (mean 10; standard deviation 3). Pairwise differences between tests and domains (Wilcoxon). *p < 0.05; **p < 0.01. Cronbach’s alpha = 0.821.

The scores for communication are particularly low, indicating significant difficulties in relation to communication skills. In this regard, participants’ performance in the receptive communication sub-test was found to be appreciably lower than in those for expressive and written communication.

Regarding daily living skills, low scores in the sub-tests for domestic and community skills indicate mild functioning problems in relation to housework and community interaction. However, the deficits observed in relation to self-care and personal management activities indicate more pronounced limitations in the personal sub-domain.

In the socialisation category, participants’ interpersonal skills were found to be adequate for them to interact socially. By contrast, they showed a low level of ability in relation to participation in leisure activities, and difficulty coping with everyday challenges. The results highlight the need for specific support and measures in relation to leisure and coping in order to improve subjects’ everyday functioning in these areas and quality of life.

The results reveal a significant correlation between interpersonal skills and autonomy (Table 6), indicating that athletes with a higher perception of autonomy in relation to Más Que Tenis activities tend to have more effective interactions with other people. The reason for this may be that autonomy improves self-confidence and the ability to manage social interactions effectively and independently.

**Table 6:** Correlations between basic psychological needs (autonomy, competence, relatedness) and Vineland-3 scale domains (communication, daily living skills, and socialisation) and MABC checklist (BOC-a: stable-predictable environment; BOC-b: dynamic-unpredictable environment).

	Basic psychological needs		
	Autonomy Rho *(p)*	Competence Rho *(p)*	Relatedness Rho *(p)*
**Communication**	0.067	0.030	0.131
Receptive	0.285*	0.219	0.211
Expressive	0.144	0.199	0.239
Written	0.171	0.105	0.189
**Daily living skills**	0.141	0.164	0.005
Personal	0.025	0.101	0.090
Domestic	0.167	0.211	0.017
Community	0.210	0.225	0.169
**Socialisation**	0.261	0.248	0.223
Interpersonal	0.284*	0.270	0.232
Play and leisure	0.237	0.189	0.250
Coping	0.362*	0.330*	0.361*
**BOC-a**	−0.288*	−0.326*	−0.248
**BOC-b**	−0.555***	−0.480***	−0.513***

Spearman’s rho: *p < 0.05; ***p < 0.001.

A significant correlation was also observed between BPN and coping skills, indicating a meaningful relationship between adaptive behaviour, resilience and effective everyday stress management, and greater satisfaction of BPN through sport (Nota et al., 2007; Viñas-Poch et al., 2014). The reasons for this may be, firstly, that greater perceived autonomy comes from having the ability to make decisions and take action according to one’s own aims and values, which in turn strengthens the ability to cope with challenges effectively. Likewise, satisfaction of the need for competence in different areas can give subjects the skills and self-confidence they need to manage adverse situations constructively. Finally, athletes with a greater perception of relatedness in a sporting context may have greater access to the social support resources they need to cope with emotional and practical challenges in their daily lives.

In the MABC-2 observation checklist, participants performed below the neurotypical average, indicating certain deficits in motor functioning. Athletes’ performance was observed to be better in controlled, static-predictable environments in comparison to more variable, dynamic-unpredictable ones (*p* < 0.005), and they scored higher on items related to personal autonomy (*p* < 0.001), indicating better motor skills in relation to everyday personal self-care.

Athletes’ family members reported that their motor difficulties did not have a significant impact on any of the following functional areas: educational/occupational, recreational, social, and self-esteem (Friedman: *χ*^2^ = 8.12; *p* > 0.05). This may be due to the implementation of effective adaptations or support strategies to mitigate the subjects’ functional deficits in these areas.

The correlations in this regard highlight the role of the athletes’ environment in relation to satisfaction of BPN through PA (Table 6). The correlation between satisfaction of BPN through a sports programme involving changing and uncertain situations of play and motor performance in dynamic, unpredictable contexts is much more intense than that observed between satisfaction of autonomy and competence and stable, predictable contexts; no correlation was observed between the latter context and relatedness.

These results offer insight into the specific needs and strengths of athletes with ID in terms of their motor and functional skills in different contexts.

### 3.4 Satisfaction of basic psychological needs by gender

As mentioned in the introduction, gender is one of the key factors traditionally examined in relation to satisfaction of BPN. For this study, a Mann–Whitney U test was run to assess the differences between men and women in relation to the variables studied. As Table 7 below illustrates, the male participants in the Más Que Tenis programme scored significantly higher than their female counterparts in relation to satisfaction of all three BPN. By contrast, no significant differences were observed in relation to satisfaction with life or physical self-concept, though female athletes scored 10 points higher in the latter than their male counterparts.

**Table 7:** Basic psychological needs, physical self-concept and life satisfaction.

Variables		Gender				*Z*
		Male (*n* = 34)		Female (*n* = 16)		
		*M*	SD	*M*	SD	
BPNES	Autonomy	4.22	0.69	3.79	0.86	−2.022*
	Competence	4.39	0.64	3.89	0.85	−2.165*
	Relatedness	4.62	0.44	4.01	0.81	−2.913**
AF5	Physical SC	55.77	39.68	66.44	29.89	−0.315
SWLS	Satisfaction with life	4.24	0.82	3.93	0.86	−1.310

Comparison between male and female athletes. Mann–Whitney U test (*n* = 50).

BPNES, Basic Psychological Needs in Exercise Scale; AF5, Physical self-concept based on AF5 Self-concept Scale; SWLS, Satisfaction With Life Scale. *p < 0.05, **p < 0.01.

## 4 Discussion

The main purpose of this study is to assess the contribution of the Más Que Tenis recreational sports programme to the satisfaction of BPN among athletes with ID, and to analyse the relationship between satisfaction of BPN and physical self-concept, motor behaviour, satisfaction with life, adaptive behaviour and gender.

The high scores reported for all three BPN, and relatedness in particular, contrast with other studies in this area (Komenda et al., 2022), where satisfaction achieved through sports programmes for athletes with ID is highest in relation to competence, and less significant in relation to autonomy and relatedness. However, it is important to note that the data for these studies are often obtained through events or competitions related to the Special Olympics and thus involve more competitive athletes with ID (Asunta et al., 2022; Hassan et al., 2012; McConkey and Menke, 2020), whereas this research focuses on a heterogeneous group of athletes with ID with varying levels of sports proficiency and competitive interest, and a sports programme whose main function and benefit is socialisation rather than competition (Varela et al., 2023). As well as limiting the scope of the research, this more competitive profile of the participants and research context accounts for the higher levels of satisfaction reported in relation to competence and the greater focus on competence by coaches.

One of the main contributions of this study is the finding that athletes with ID with a more basic level of athletic ability and a purely recreational interest in sport report satisfaction of all three BPN at a similarly high level based on their participation in the Más Que Tenis programme, with relatedness (rather than competence) scoring highest of the three. This finding is in line with the conclusions of other studies in adapted sports settings, in which sport has been shown to provide opportunities for socialisation (Shapiro and Martin, 2014), as well as personal and social growth, and a sense of belonging to a community and sharing a set of common goals (White et al., 2017).

Satisfaction of BPN, personal growth and satisfaction with life are closely related to self-concept, an area which has received little research attention in relation to people with mild to moderate ID (Reel et al., 2013). Positive and negative self-perception are directly related to feelings of general satisfaction, body satisfaction and self-esteem (Perrotta, 2011; Reel et al., 2013). While people with ID generally report lower levels of self-esteem than the rest of the population, participation in sports experiences has been shown to have a positive effect on both self-concept and sports competence (Pan and Davis, 2018). The participants in this study declared that they were good at sport and satisfied with their body image. However, they were also critical in their assessment of their attractiveness and their efforts in relation to physical self-care. In keeping with previous studies, positive self-concept was found to correlate with life satisfaction, with participants reporting high levels of satisfaction with their lives and personal circumstances. The findings in relation to the association between life satisfaction and BPN diverge from those reported elsewhere (Prado-Botana et al., 2023), however, with correlations observed between physical self-concept and autonomy and competence, but less noticeable with relatedness.

In relation to the coping skills assessed through the Vineland-3 Adaptive Behaviour Scale, the study’s findings indicate a deep connection between executive functions (EFs) and skills such as problem solving, impulse control and the ability to behave appropriately in different situations. While coping skills refer to how well a person manages stress, frustration and other emotions, EFs are defined as the mental abilities essential for carrying out effective, creative and socially acceptable behaviour (Erostarbe-Pérez et al., 2021), and include cognitive flexibility, inhibitory control, and planning and organisation. According to Erostarbe-Pérez et al. (2021), all EFs correlate significantly with general intellectual capacity, a claim confirmed by recent studies demonstrating the relationship between intelligence and working memory, cognitive flexibility and inhibitory control among adults with ID (Biesmans et al., 2019; Schuchardt et al., 2010; Memisevic and Sinanovic, 2014). Cognitive flexibility is the mental ability to adapt to changes and find new solutions to problems; athletes with high cognitive flexibility have the ability to change strategy when a coping technique is not working, e.g., by modifying a game plan in response to an unexpected situation (Diamond, 2013; Vestberg et al., 2012). Inhibitory control is the ability to control urges and inappropriate behaviour in situations of stress; athletes with good inhibitory control can resist the temptation to react impulsively and opt instead for a more appropriate response, such as staying calm and avoiding aggressive responses when faced with provocation from an opponent (Danielsson et al., 2010). Finally, planning and organisation allow people to anticipate challenges and develop strategies to deal with them effectively (Hartman et al., 2010); athletes with good planning and organisation skills have the ability to formulate plans to deal with different scenarios, such as a routine to prepare mentally before a competition (Lifshitz et al., 2007; Wehmeyer and Garner, 2003).

Among athletes with ID, the relationship between BPN, coping skills and EFs is complex and multifaceted. Satisfaction of BPN correlates intensely with motor behaviour in dynamic, unpredictable environments, such as Más Que Tenis tennis classes. Numerous studies also show that satisfaction of BPN leads to greater wellbeing and motivation among athletes with ID (Shangraw, 2017; Shogren et al., 2019), which are in turn linked with greater self-regulation and inhibitory control.

The more autonomy athletes perceive, the more in control they feel of their own actions, routines and decisions, which in turn improves their ability to inhibit inappropriate responses and stay focused on objectives (Shangraw, 2017; Shogren et al., 2019). Furthermore, autonomy improves athletes’ planning and decision-making skills by providing them with opportunities to develop their ability to assess options and plan activities.

Vestberg et al. (2012) analysis of how EFs predict success in soccer shows that players with greater cognitive flexibility are more effective when it comes to changing strategy and adapting to new circumstances. Greater sports competence not only increases cognitive flexibility, therefore, but also makes athletes more adaptable to changes and challenges, an ability that is essential in dynamic contexts such as that of a tennis class, where play is unpredictable. Satisfaction of the need for competence is also positively associated with pleasant emotions and biopsychosocial experiences perceived as functional for performance (Robazza et al., 2023).

In relation to the need for relatedness, positive social relationships have been shown to be a source of emotional support (Shogren et al., 2019), which can lead to improved emotional regulation and inhibitory control. The support of peers and coaches helps athletes to manage stress and negative emotions more effectively, and to respond in such situations in a more controlled, less reactive manner. The collaboration and effective communication components of positive social interactions in a sporting context also help to strengthen problem-solving ability by providing athletes with a more structured, strategic approach to dealing with challenges. Recent research shows that satisfaction of athletes’ need for relatedness and autonomy is related to the experience of pleasant emotions and functional biopsychosocial states based on adaptive emotional regulation (Robazza et al., 2023).

Despite the lack of research on the role of gender in relation to satisfaction of BPN among athletes with and without ID (Nieves, 2021; Rodrigues et al., 2019), the evidence indicates that in sporting contexts, as elsewhere, gender is a predictive factor of satisfaction of BPN, with female subjects scoring lower than male (Gómez-López et al., 2021; Moreno-Casado et al., 2022; Prado-Botana et al., 2023; Hu et al., 2023). In this study, the influence of gender was most pronounced in relation to satisfaction of the need for relatedness. The fact that the sample for the study was more than two thirds male may account for this low perception of satisfaction of the need for socialisation among the remaining one third (1–2 females per school).

Nevertheless, the fact that satisfaction scores for female participants were significantly lower than those of their male counterparts across all three BPN indicates that the Más Que Tenis recreational sports programme is failing to meet the needs of female athletes more generally, resulting in reduced motivation to participate, increased likelihood of abandonment, and the loss of opportunities for personal growth and development through PA. Those in charge of the programme should be mindful of this gender difference, and take targeted measures to respond more effectively to the needs of female athletes with ID.

The results of the study have important practical implications for professionals. First, professionals should pay special attention to the satisfaction of autonomy, as although participants feel that the exercises align with their interests, their perception of autonomy decreases when they are unable to choose how to perform them. This suggests the need to incorporate strategies that allow participants to make decisions about how to engage in activities, thereby promoting greater independence. Second, there is an emphasis on creating a supportive environment where participants can develop physical skills and feel part of a group. This underscores the educator’s role in fostering positive interactions and building trust among participants. Finally, the importance of a comprehensive approach that focuses not only on physical development but also on emotional and social growth through sports is highlighted.

### 4.1 Limitations and future research

As in all studies, certain limitations must be acknowledged. The first of these is the heterogeneous nature of the sample, which comprised athletes with ID of different ages and intellectual level, with different aetiologies of cognitive deficit. It is important to recognise that using convenience sampling limits the generalisation of the results to the entire population with intellectual disabilities in Spain. This limitation is due to logistical and accessibility factors for participants; however, it does not invalidate the fact that the analyses conducted provide valuable information for future research. In subsequent studies, it would be advisable to conduct random sampling or use more representative sampling techniques to validate and expand these findings.

Another limitation is the absence of a control group of subjects with ID who do not participate in sports activities, which would allow us to compare the data for the different variables studied between athlete and non-athlete groups.

Similarly, a sample group representative of the whole spectrum of sports proficiency, from beginner to high performance, would allow us to assess satisfaction of BPN among athletes with ID by sporting level.

The significance of the differences detected in Satisfaction of BPN by gender section should be interpreted with caution due to the large difference between the size of the two groups compared (many more males than females).

Finally, the relationship between satisfaction of BPN and executive functions has been inferred from the results of the Vineland-3 scale assessment of coping skills. More specific, validated testing is necessary in order to measure executive function more precisely.

With regard to future avenues of research, it would be interesting to implement longitudinal studies with athletes with ID to observe the influence of participation in recreational sports programmes on satisfaction of BPN and personal wellbeing over time, and to assess its long-term impact.

Likewise, it would be useful to examine the role of social environment (peers, coaches, family members) on satisfaction of BPN and personal wellbeing, and how different types of social support influence motivation and persistence in PA among people with ID.

In relation to self-determination theory, more research is needed on the motives for participating in sport among people with ID, and how those motives relate to BPN.

Another area of huge interest and future research is the comparative satisfaction of BPN through sports programmes among men and women with ID.

Finally, research on Special Olympics athletes in Spain still has a long way to go to catch up with the literature in this area in other countries.

## 5 Conclusion

The study revealed a high level of satisfaction of BPN among participants in the Más Que Tenis programme, with satisfaction of the need for relatedness ranking higher than autonomy and competence, in contrast to studies involving high performance athletes with ID, in which satisfaction is highest in relation to competence. This allows us to confirm our working hypothesis number 1 (H1).

Most participants were found to have a positive physical self-concept and a high level of satisfaction with their life in general. Satisfaction of BPN correlated positively with life satisfaction, and more moderately with physical self-concept, with significant values in relation to autonomy and competence, but not relatedness. According to this information, the study’s second hypothesis (H2) is confirmed.

The results showed a significant correlation between interpersonal and coping skills and satisfaction of the need for autonomy, and highlighted the importance of both sets of skills to ensure effective adaptive functioning. The interconnections identified between executive functions (cognitive flexibility, inhibitory control, and planning) and coping skills show how the Más Que Tenis programme can help athletes to manage both their stress and the demands of sport more effectively. The dynamic, unpredictable environment created by the programme resulted in an intense correlation between satisfaction of all three BPN and motor performance in situations of change and uncertainty. According to these data, the third working hypothesis (H3) is partially confirmed since the positive relationship between BPN satisfaction and motor behaviour occurs mainly when there is coherence between the characteristics of the sports context (Más Que Tenis programme) and the context of everyday life. Most of the skills developed in the programme occur in contexts of open sports practise and uncertainty, so there is a strong correlation between the satisfaction of the BPN and daily motor behaviour in dynamic and unpredictable situations. This correlation is much lower or non-existent when the daily life situations are closed and without uncertainty.

Regarding the role of gender as a predictor of satisfaction of BPN, the study found that male participants perceived higher levels of satisfaction across all three BPN, indicating that the programme is less effective in meeting the needs of female athletes with ID. Working hypothesis 4 (H4) is thus confirmed.

As an additional conclusion, the findings of the study confirm the universality of the BPN model by showing similar applicability across neurotypical and neurodivergent populations.

Overall, the study shows that the Más Que Tenis recreational sports programme not only improves the tennis skills of athletes with ID, but also contributes to their personal and neuropsychological wellbeing by satisfying BPN, promoting a positive social environment, and developing their coping skills and everyday functioning.

## Data Availability

The raw data supporting the conclusions of this article will be made available by the authors without undue reservation.
